# Adolescent perspectives on depression as a disease of loneliness: a qualitative study with youth and other stakeholders in urban Nepal

**DOI:** 10.1186/s13034-022-00481-y

**Published:** 2022-06-23

**Authors:** Syed Shabab Wahid, Katherine Ottman, Jyoti Bohara, Vibha Neupane, Helen L. Fisher, Christian Kieling, Valeria Mondelli, Kamal Gautam, Brandon A. Kohrt

**Affiliations:** 1 Department of International Health, Georgetown University, Washington, DC USA; 2 Division of Global Mental Health, George Washington University, Washington, DC USA; 3 Transcultural Psychosocial Organization Nepal (TPO Nepal), Baluwatar, Kathmandu, Nepal; 4 Social, Genetic & Developmental Psychiatry Centre, Institute of Psychiatry, Psychology and Neuroscience, King’s College London, London, UK; 5 ESRC Centre for Society and Mental Health, King’s College London, London, UK; 6 Department of Psychiatry, Universidade Federal do Rio Grande do Sul and Child & Adolescent Psychiatry Division, Hospital de Clínicas de Porto Alegre, Porto Alegre, RS Brazil; 7 Department of Psychological Medicine, Institute of Psychiatry, Psychology & Neuroscience, King’s College London, London, UK; 8 National Institute for Health Research Mental Health Biomedical Research Centre, South London and Maudsley NHS Foundation Trust and King’s College London, London, UK

**Keywords:** Depression, Loneliness, Social isolation, Qualitative research, Adolescence, Nepal

## Abstract

**Background:**

There is a lack of research on the adolescent experience of depression in low- and middle-income countries. Criteria derived from research conducted primarily among adult Western populations inform current diagnostic standards for depression. These clinical categories are often used without exploration of their relevance to adolescent experience. Also, reliance on these categories may overlook other symptoms of depression that manifest in non-western settings. Cross-cultural qualitative work with adults in non-Western settings has suggested some differences with experience of depression and symptoms that are most relevant to service users. Research into adolescent experiences of depression is warranted to inform the development of effective interventions.

**Methods:**

Qualitative interviews were conducted in Nepal with adolescents with depressive symptoms (n = 9), healthy adolescents (n = 3), parents (n = 6), teachers (n = 10), social workers (n = 14), primary (n = 6) and mental (n = 6) healthcare providers, and policymakers (n = 6). Two focus groups were conducted with parents (n = 12) of depressed and non-depressed adolescents. Data were analyzed according to the framework approach methodology.

**Results:**

Loneliness was the hallmark experience that stood out for all adolescents. This was connected with 5 other clusters of symptoms: low mood and anhedonia; disturbances in sleep and appetite, accompanied by fatigue; irritability and anger; negative self-appraisals including hopelessness and self-doubt; and suicidality. Adolescents distinguished depression from other forms of stress, locally referred to as *tension*, and described depression to involve having “deep *tension*.” Perceived causes of depression included (1) Family issues: neglectful or absent parents, relationship problems, and family discord; (2) Peer relationships: romantic problems, bullying, and friendship problems; and (3) Social media: social comparison, popularity metrics, cyberbullying, and leaking of personal information.

**Conclusions:**

Consistent with other cross-cultural studies, loneliness was a core element of the adolescent experience of depression, despite its absence as a primary symptom in current psychiatric diagnostic classifications. It is important to note that among youth, symptoms were clustered together and interrelated (e.g., sleep and appetite changes were connected with fatigue). This calls for the need for more cross-cultural qualitative research on experience of depression among adolescents, and potential for modification of diagnostic criteria and prevention and treatments to focus on the experience of loneliness.

**Supplementary Information:**

The online version contains supplementary material available at 10.1186/s13034-022-00481-y.

## Introduction

Adolescence is a pivotal period of human development (often defined as occurring between 10–24 years of age) characterized by important physical and emotional changes that impact pathways of well-being throughout life [1]. Developmental trajectories occur through complex interactions of key relationships and broader contextual and cultural factors [2]. These can combine to foster healthy development and build resilience or manifest risks to well-being [3, 4].

In particular, adolescence represents a time of increased vulnerability for developing depression. The incidence of depression peaks during this period and can continue as a lifelong burden if left unaddressed [5, 6]. Globally, depression is one of the leading causes of illness and disability among adolescents [7] and is especially problematic as it is characterized by high recurrence rates and poor health outcomes [8]. Additionally, suicide risk is magnified among depressed adolescents globally, and represents the second leading cause of death in this population according to 2015 estimations by the World Health Organization [9]. Accordingly, addressing the global burden of adolescent depression becomes highly salient in efforts to achieve Sustainable Development Goal 3 [10] which aims “to ensure healthy lives and promote well-being for all at all ages.”

Current models of adolescent depression, such as those outlined in the *Diagnostic and Statistical Manual of Mental Disorders* (DSM), are conceptualized as presenting core symptoms similar to symptom profiles observed among adults [11]. These profiles are derived from research conducted predominantly in Western, Educated, Industrialized, Rich and Democratic (WEIRD) countries [12, 13]. This “etic” approach is problematic for three reasons. First, symptom profiles derived from WEIRD societies do not account for heterogeneity of the experience of depression around the world [14], and may not adequately reflect the experiences most relevant to populations in diverse global contexts [15]. Second, recent research indicates that adolescent depression may consist of varying clusters of symptoms that are indeed different from adults [16], which manifest via different pathophysiological mechanisms for adolescents as compared to adults [17]. Finally, a focus on adolescents living in non-WEIRD nations is necessary because 90 percent of the world’s young people live in low- and middle-income countries (LMIC) [18], and inclusion of youth perspectives from LMIC is crucial in informing a more holistic understanding of adolescent depression.

Considering these multiple factors, there have been calls for research that go beyond standard biomedical DSM diagnostic criteria to develop an updated “emic” understanding of depression that is grounded in the actual experience of adolescents [16, 19] and within the unique ecological conditions of cultures across the world [14], rather than solely extrapolating from WEIRD adult symptom profiles. Qualitative research can elicit detailed accounts of the experience of depression and culturally salient idioms of distress as contextualized within broader sociocultural conditions, and therefore, contribute to addressing these gaps in the literature.

The qualitative literature surrounding adolescent depression in non-WEIRD nations is limited but growing, and indicate symptomatology that may indeed lie beyond the limited bracketing of DSM criteria, and may be manifested via complex interactions with contextual factors. In Brazil, Viduani et al. [20] finds social isolation to be the defining feature of adolescent depression, among other non-DSM symptoms such as emptiness and poor academic performance. Similarly, Kok et al. [21] report desiring connectedness with others to be highly salient among depressed young people in Malaysia. In a recently conducted qualitative study of adolescent depression in rural Nepal [22], respondents highlighted the salience of interpersonal problems such as grief, dispute, role transition, and social isolation as well. Emotional and physical abuse were also discussed, especially in parental relationships. Among depressed Arab adolescents, Dardas et al. [23] find the presence of non-DSM symptoms such as crying a lot, loneliness, bad school performance, somatic complaints, elevated stress, shame, and pessimism. Finally, in a study with HIV-positive adolescents with depression in Zimbabwe participants reported a sense of isolation and rejection, and a desire to be important or to matter to their loved ones as the most important aspects of their experience [24]. However, substantial knowledge gaps about adolescent depression persist across many LMICs, which can hinder culturally salient and age-appropriate care provision.

Accordingly, in this study, we used qualitative methods to examine the experience of depression from depressed adolescents in Nepal, an LMIC in South Asia. We also examined notions about adolescent depression from other stakeholders who are key actors in the ecological spaces depressed adolescents inhabit and participate in.

## Study setting

The pertinent need for this type of research is reflected in Nepal, a lower-middle income country with a large youth population. Adolescents in Nepal grew up experiencing humanitarian emergencies, including a decade-long civil war and multiple environmental disasters, among other socio-cultural risk factors for mental disorders [25–27]. In one study, 14 percent of the adolescent population in Nepal reported at least one psychosocial problem [28].

## Methods

This qualitative study is part of a larger research portfolio being implemented by the Identifying Depression Early in Adolescence (IDEA) research consortium [29]. The study design, recruitment and sampling, and research team characteristics have been described in detail in the study protocol, which has been published elsewhere [30]. The pragmatist research paradigm which is focused on “what works” guides this study as our ultimate goal was to inform better detection and treatment of adolescent depression in Nepal [31]. We used the framework approach to guide overall data analysis [32]. Key-informant interviews (KII) and focus group discussions (FGD) with adolescents, parents, educators, health workers, social workers, and policymakers were conducted. The interview guide is available in an additional file (see Additional file 1). Our adolescent sample (n = 12) included seven individuals with a history of depression, two individuals who were referred to psychological counseling exhibiting clinical signs of depression, and three individuals with no history of mental health conditions. Non-adolescent stakeholders included parents (n = 18: KII = 6; FGD = 12), teachers (n = 10), social workers (n = 14), primary healthcare providers (n = 6), mental healthcare providers (n = 6), and policymakers (n = 6). Respondents were selected on the rationale that they either had direct experience of adolescent depression or were key actors in shaping adolescent lives. We utilized a purposive method in recruiting adolescents with experience of depression and parents of depressed adolescents. We also purposively recruited policy officials at relevant ministries (health, education, and social services). We utilized a convenience method for sampling all other respondents. Recruitment involved face-to-face efforts, and email and telephone solicitation. Depressed adolescent respondents had been diagnosed with depression via clinical intake interviews conducted by experienced Nepali psychiatrists who are intimately familiar with the Nepali cultural context. Qualitative interview and group discussion guides were structured according to Kleinman’s explanatory model framework of mental illness and Engel’s biopsychosocial risk factors approach [33, 34]. The key topics were the experience of adolescence, the experience of adolescent depression, adolescent coping strategies, and societal perspectives of depression. The role of various relevant stakeholders in the identification of depression was explored as well. The qualitative interview guides were translated from English by bilingual Nepali mental health researchers trained in transcultural research methods at the Transcultural Psychosocial Organization Nepal (TPO Nepal) and reviewed by a co-investigator of the IDEA project who is a senior Nepali researcher. We conducted six preliminary interviews to pilot the guide and subsequently adjusted the instrument for cultural and contextual considerations.

Interviews were conducted at several locations in the greater Kathmandu area including a non-profit organization dedicated to psychosocial well-being, partner hospitals, and respondent offices. Interviews were conducted in Nepali or English, according to the preference of the respondent, by three female study authors (KO, JB, and VN) and a male research assistant, who are trained in cross-cultural psychiatry research methods. The interviews were conducted with one interviewer and one observer from the research team and lasted 45 min on an average. Qualitative data from the KIIs and FGDs were translated from Nepali to English. An a priori theory-informed codebook was initially created which was subsequently modified to include inductive codes and categories identified in the data. The finalized codebook was applied to the full dataset using NVivo version 12 [35]. A team of five researchers from Nepal and the United States coded the data. Further details of the entire research team are included in an additional file (see Additional file 2). We established inter-rater reliability by achieving a Cohen’s kappa statistic of 0.7 within the research team, which represents substantial agreement [36]. To increase rigor, we used the constant comparison approach during coding by comparing newly coded sections with previous segments across the coding team, to ensure consistent application of codes across the dataset [37]. We utilized the principles of ‘information power’ proposed by Malterud et al. [38] to determine data saturation, namely the criteria of high-quality dialogue with interlocutors, narrow focus of the aim, and high specificity of the experience (adolescent depression) under investigation, all of which endorse relatively smaller sample size requirements for saturation [38]. After coding was completed, we executed code queries in NVivo stratified by adolescents and other stakeholder groups, for each code. Code summaries were written to synthesize respondent narratives by stakeholder type to allow comparison across different stakeholder perspectives. The study followed the COnsolidated criteria for REporting Qualitative research (COREQ) guidelines (see Additional file 3).

## Results

The demographic characteristics of the respondents are provided in Table 1. We organize and present the results with a primary focus on adolescent experiences which are supplemented by adult stakeholder perspectives.

**Table 1:** IDEA Nepal Qualitative Study Respondent Characteristics (n=72)

Adolescents (n = 12)	*n*	%
Age (in years)		
10–13	2	16.7
14–18	3	25
19–24	7	58.3
Gender		
Female	10	83.3
Male	2	16.7
Marital status		
Married	0	0
Unmarried	12	100
Depression status		
Depressed	7	58.3
Referred to counseling	2	16.7
Not depressed	3	25
Caste		
*Brahmin*	7	58
*Janajati*	4	33.3
*Chhetri*	1	8.3

Non-adolescents (n = 60)		
Age (in years)		
20–29	10	16.7
30–39	25	41.7
40–49	12	20
50 and over	8	13.3
Not reported	5	8.3
Gender		
Female	28	46.7
Male	32	53.3
Marital status		
Married	47	78.3
Unmarried	10	16.7
Divorced/widower	2	3.3
Not reported	1	1.7
Caste		
*Brahmin*	31	52
*Janajati*	20	33.3
*Chhetri*	6	10
Not reported	3	5
Occupation		
Teacher	4	6.7
Other school worker*	6	10
Social worker	14	23.3
Psychiatrist/psychologist	6	10
Other health worker	6	10
Policy maker	6	10
Parent/caregiver	18	30

*All other non-teachers who work within the school environment; this includes headmasters, school nurses, and school counselors

## The experience of adolescent depression

“Depression is a condition where a person is not healthy mentally or emotionally.” *– 20-year-old depressed female (NP-006)*

**Fig. 1:**
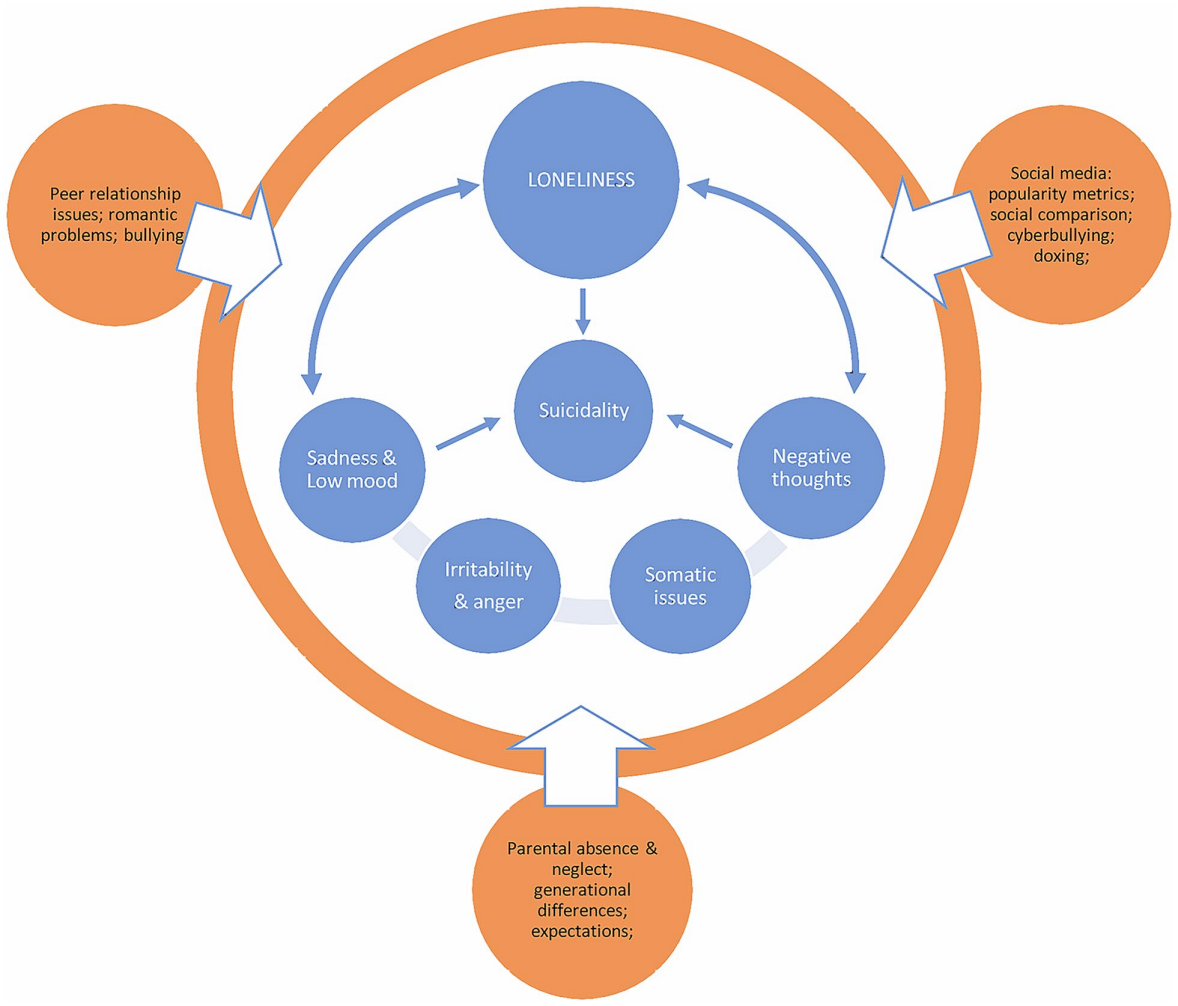
The experience of depression and its causes as described by Nepali adolescents

Key symptoms of depression described by adolescents included a core band of six clusters of experience: (1) loneliness; (2) sadness, low mood, and anhedonia; (3) irritability and anger; (4) somatic problems; (5) negative thoughts, rumination, and worries; and (6) suicidality. The connections between these experiences and perceived causes of depression, as described by respondents, are visually summarized in Fig. 1.

### Loneliness

Loneliness was the most salient aspect of depression described by adolescents. Loneliness included powerful feelings of wanting to be alone, social withdrawal away from family and friends, and the inability to open up and share one’s feelings. Loneliness was described as reinforcing other core symptom clusters of depression, which in turn reinforced loneliness, in a vicious cycle, as portrayed in Figure-1. Adolescents, both depressed and healthy, described how depressed adolescents prefer being alone. Depressed adolescents did not necessarily report lacking supportive or meaningful friendships. Instead, they narrated how depression caused them to wish to be alone, despite having friends:

"During that time [depression], I disliked meeting friends. Disliked as in I was happy to know I have friends, but I didn’t feel like doing anything. I did not feel like going and talking to them." – *23-year-old male recently recovered from depression (NP-003)*

This adolescent further elaborated how feelings of sadness and low self-esteem fueled their desire to stay away from his friends:

"I used to be sad all the time and did not like meeting up with people. If anyone asked to hang out, the feeling of emptiness and uncertainty about what I would have to talk about would be there… I did not like to face anyone, not even close friends, or anyone! So, [when I did run into friends] I kept going with the flow and kept trying to move on…. I would just wait for the conversation to end and try to get back to my own world as far away [from others] as possible." - *23-year-old male recently recovered from depression (NP-003)*

Others described how depression led to a slow alienation from their friends and family, leading to deterioration of existing friendships:

“Yes, [friends avoid me]. I was a fun person before. I had *tension* earlier, but I wasn’t depressed. When I was a fun person, they used to feel good around me. After I started getting pressure from the family [during depression], I began to stay silent; they [friends] started getting bored being with me and everybody kept leaving me.” *– 13-year-old female recently referred to care (NP-035)*

Adolescents described how they could not open up to others to share their feelings. These feelings would mostly be suppressed and kept to themselves, while presenting a normal appearance to family or friends:

“I wanted to live alone, did not want to talk to others, used to get angry easily, tears used to roll down my face the moment I was alone.” - *13-year-old female recently referred to care (NP-034)*

“I get stressed with small things. I go to the toilet and cry alone. I put on a fake smile in front of mother. I don’t want to share with anyone.” – *13-year-old female recently referred to care (NP-035)*

One adolescent even attributed the inability to share her problems as one of the causes of depression itself:

“I think depression is caused due to one being unable to express themselves.” *20-year-old depressed female (NP-006)*

Adolescents’ desire to be alone and unwillingness or inability to open up and share their problems were corroborated by other study participants as well. These included schoolteachers and school counselors, social workers, primary healthcare providers, and parents of depressed adolescents. One schoolteacher shared:

“Speaking about mental health, [adolescents] want to stay alone. They cannot share their feelings openly in front of others…They feel uncomfortable [sharing] even though it is natural. Because of that, they feel awkward. So, the problems are them staying alone and over-thinking about things, not wanting to be among their friends. As such, I see [depressed adolescents] spending time by themselves mostly.” – *30-year-old schoolteacher (NP-045)*

Interestingly, only one mental health care provider (out of six) raised loneliness as an important aspect of adolescent depression, while the others primarily discussed symptoms from DSM criteria.

### Low mood, sadness, and anhedonia

Most depressed adolescents described anhedonic-type experiences of losing interest in activities that were previously considered to be enjoyable. One respondent shared:

“Things you like doing everyday become things you don’t want to do anymore.” – *15-year-old depressed female (NP-039)*

Strong and persistent sadness and emptiness were almost universally reported. One adolescent shared her experience of depression onset before being connected to care:

“I used to be sad all the time and I did not share it with anyone, not even my sister with whom I used to live. When I looked at people being happy and laughing, I used to wonder how they can laugh like that. I used to think that I will never experience a time when I would also be laughing like those people. I did not know it was depression. If I knew, I would have visited a doctor. That phase slowly passed, but the pain still remained somewhere in my heart. Then afterwards, I used to feel deep pain and sadness sometimes and sometimes I used to feel normal. Gradually the pain began to increase, and I could not sleep.” – *20-year-old depressed female (NP-006)*

All other stakeholder groups endorsed sadness and low mood as a definitive aspect of depression. A primary care provider shared:

“Sadness also comes [with depression]. Persistent sadness and pervasive sadness. One is always sad and is sad from inside. He may look happy from the outside but in reality, he is sad on the inside.” *– 28-year-old male primary health care center provider (NP-014)*

### Somatic problems: appetite issues, sleep disruptions, and chronic fatigue

Adolescents strongly endorsed experiencing somatic issues due to depression. These consisted of disrupted patterns of sleep, loss of appetite, and tiredness. One participant connected his experience of chronic low energy with losing interest in normal activities:

“Some people have difficulty sleeping [during depression] but I had sleepiness all throughout the day. It was very difficult for me to be out of bed for even one and a half hours. Honestly, other than going to classes or something similar, I used to stay bed ridden. Loss of appetite also happens for some [who are depressed] …I also had appetite loss but used to force myself to have food…due to energy loss I lost interest in doing those activities which used be normal previously. Energy used to go down to an extreme low and it felt very difficult to come out of that state.” – *23-year-old male recently recovered from depression (NP-003)*

### Irritability and anger

Adolescents universally endorsed feelings of irritability and flashing bursts of anger as a common aspect of depression. Irritability at small things and especially towards family was cited as a reason for adolescents wanting to be left alone:

“My parents said that I used to get angry all the time [during depression]. It was about almost a year or half back and I am now getting better, and I don’t behave like that as much. The characteristics [of depression] include aggressive and irritable behavior, wanting to stay alone, not socializing with family, and acting aggressively with parents when they want us to stay with them and put our mobile phones away for a while. I used to do these things.” – *18-year-old depressed male (NP-011)*

This adolescent elaborated on his anger and linked it to self-harm:

“People may harm themselves because of the anger they have for others; for example, if I am angry with my parents as they always ask me to stop doing things I am doing, then I might harm myself to express the anger I have towards my parents.” – 18-year-old depressed male (NP-011)

Social workers, school workers, parents, and mental health care providers confirmed irritability, anger, and aggressiveness as key aspects of adolescent depression as well. One social worker shared:

“[They get] irritated in small things, fighting, or arguing continuously, being aggressive, running away from home, intimidating others, skipping school.” – *35-year-old male social worker (NP-038)*

### Negative thoughts

Depressed adolescents described a range of negative cognitive experiences during depression. Some described maladaptive thought patterns of persistent hopelessness and overall negativity. One respondent shared:

“I used to always have a feeling of hopelessness and negativity [during depression]. I used to see everything negatively. But now the situation is not the same. Now I have a feeling that ‘it will pass.’” – *20-year-old female undergoing treatment for depression (NP-043)*

Others described chronic self-doubt, magnified negative thoughts, perceptions about not being able to meet expectations of oneself or others, and believing that one's life circumstances could not be changed. One depressed respondent described his thought patterns:

“I feel hesitant while doing something. I feel like I can’t do anything and feel like I don’t know how to do things. I also feel like what I am doing might be wrong…I get thoughts like, ‘I can’t study, like I can’t do it, that I just forget things…and if I can’t study, what will be my future?’ I can’t do anything… that I am useless!” – *15-year-old depressed female (NP-039)*

Adult stakeholders also endorsed these types of negative thinking as being present in adolescents with depression. One school worker shared:

“From their thoughts; if we talk to them for a while, then the things that they talk about lead towards sadness. They say they will not be able to trust anyone. They do not give themselves any self- importance. They feel that there is no need for them to live anymore. They feel that there is nothing that they can do.” – *27-year-old female schoolteacher (NP-026)*

### Suicidality

Suicidality was discussed as an outcome of acute depression where increasing and unaddressed sadness, worries and loneliness were said to eventually culminate in suicidal thoughts and attempts at self-harm. Most depressed adolescents strongly endorsed experiencing thoughts about suicide and self-harm at some point during depression. Some shared that they had attempted suicide or engaged in attempts at self-harm. Feelings of increasing disconnection from family and friends were cited as a major reason for suicidal thoughts to arise by respondents. An adolescent who had recently been referred to psychological care described a conflicted state of struggle against recurrent feelings of wanting to die.

“Yes, I had attempted [suicide] several times thinking all people take me [negatively]. When I tried [to kill myself], I used to look at myself in the mirror. My eyes used to be swollen from crying. I have aspired to grow up to be a normal person since childhood. I will fulfill that no matter what. Whatever other people do or say does not matter, I will live my life – that’s how I feel. However, I also often feel like I want to die…” – *13-year-old female recently referred to care (NP-035)*

One adolescent described fractious relationship dynamics with their parents and recounted her self-harming acts before they were diagnosed with depression, and how irritability and anger at parents fueled such thoughts:

“As I didn’t know [that I had depression] before, I used to consider my [self-harming] behavior to be normal. After I got diagnosed, now I feel this behavior is childish. I had used a compass and a pencil sharpener’s blade to hurt myself. My mother says, you should not do these things, which irritates me and makes feels like hurting myself more. I don’t’ feel like receiving support!” – *14-year-old depressed female (NP-036)*

Another adolescent who was awaiting assessment, recounted a period of family strife where she had closely witnessed several aggressive fights between parents. She described intense feelings of sadness and loneliness and how these were related to suicidal thoughts:

“When I am in a lot of stress, my heart and body feel like: ‘Where to go, what to do?’ I did not want to go home on time after school, wanted to live alone, wanted to cry, sometimes felt like dying. Just like that, the feeling of dying would come; wanted to cry, wanted to live alone, did not want to return home on time so on.” - *13-year-old female recently referred to care (NP-034)*

## Distinguishing depression from other experiences

In addition to the previously described aspects of adolescent depression, adolescents and other stakeholders discussed how other experiences were often conflated with depression, or that people chose to use different terms or idioms of distress to discuss depressive symptoms. One respondent shared how they initially did not understand how to differentiate sadness from depression:

“So, about depression…before my diagnosis I thought it was just sadness but, after I was diagnosed, I understood that sadness is only one part of it. Depression could make one physically unable to do many things. If someone gets affected for more than 2 to 3 weeks, then I think we can use ‘depression’ for that. So, continuous sad mood…. [and]… functioning needs to be hampered.” – *23-year-old male recently recovered from depression (NP-003)*

Some adolescents mentioned that they did not know they were depressed until much more prominent and advanced symptoms started appearing. One depressed adolescent recalled how they did not know if their experience was something wrong for which they should be seeking help:

"I went for the treatment only after I was really struggling with depression, and I am still taking medicine for it. But I think that if I went for treatment in the initial phase of my depression then it would have been easy for me to recover… I didn’t know it was depression, if I knew, I would have visited a doctor." *– 20-year-old depressed female (NP-006)*

One adolescent described their confusion about symptoms of anxiety and conflated those with depression:

“I felt really nervous and panicked. And if someone raises their voice at me even at the small things, I feel really hurt and I over think the situations [interviewer probes on what word can be used to describe these feelings] ‘Depression’ I think… ‘anxiety?’ They say I have an attack.” – 1*5-year-old depressed female (NP-039)*

Another adolescent mentioned that the actual English word ‘depression’ is used in everyday conversation in Nepal to communicate normal and far less severe distressed states. Accordingly, for some, depression and sadness are not recognized as different things:

“The word ‘depression’ is used [to communicate that someone is depressed]. In fact, even if people are experiencing normal sadness, they will describe that as depression too. For example, let’s say an exam is coming up and there is only one week to study, then people say, ‘There isn’t enough time, I am having depression!’ So… [‘depression’ is used] in a normal way. Thus, we do not see sadness and depression as two different things.” – *20-year-old female undergoing treatment for depression (NP-043)*

This use of the word ‘depression’ by adolescents to communicate milder states of distress was corroborated by mental care providers, primary health providers and social workers. These stakeholders explained that the word ‘depression’ is used as a common term used by lay people in Nepal for all mental conditions, or for specific symptoms of clinical depression, e.g., a person may refer to sleep or appetite disruptions as “depression.” One primary healthcare provider explained how they would have to explain to some patients that “depression” is not a disease, but rather that “depressive illness is the disease.” One psychologist shared how they approach adolescent patients:

“The word ‘depression’ is quite common among adolescents because they have heard it from so many sources. We tell them: ‘[You have] depression?’ Okay. ‘Normal depression?’ Or ‘depression’ where you need help?” – *36-year-old male psychologist (NP-008)*

Quite a few respondents mentioned *tension* in relation to depression. *Tension* is a popular local idiom of distress in Nepal [39]. “Deep *tension*” or thinking about a stressor constantly was described by some respondents to lead to depression, while another respondent mentioned that depressed people “take *tension*” in everything.

“*Tension* is about normal things. Depression is caused by deep *tension*” – 1*5-year-old depressed female (NP-039)*

One adolescent shared their perceptions on depression and how it incorporated *tension*:

“Depression is very bad. Depression has stages such as difficulty, danger and so on. When in depression, we do not want to talk to others, do not want to walk, we take *tension* in everything.” *– 13-year-old female recently referred to care (NP-035)*

Others clearly differentiated *tension* to be something milder, and different from sadness or other aspects of depression. Another adolescent with no history of depression shared:

“*Tension* and sadness, that’s different. Because tension is something you take because you feel like you are in a problem — if something happens, it will affect you… an example of *tension* is like…maybe parents are thinking you should get married very soon (laughs). That is a *tension*!” *– 22-year-old female adolescent with no depression (NP-018)*

## Adolescent depression: causes

Adolescents attributed three major clusters of causes to depression. These included: (1) problems with the family, especially with parents, involving parental absence or neglect, communication breakdowns due to generational differences, and abusive family environments; (2) peer relationship problems which included issues with friends, exclusion, bullying, and romantic relationship problems; and (3) social media, which was described as an important ecological space for adolescents, but fraught with risk factors for depression including excessive social comparison, popularity metrics, compulsive use of social media, excessive online gaming, exposure to strangers on the internet, and public leaking of personal information, often in the form of sexually explicit photographs. These three causal domains were also strongly endorsed by adult stakeholders.

### Parental strife, absence, and neglect

Adolescent respondents indicated several issues concerning family that could be contributing towards depression in adolescents. Parental absence, due to parental work demands, was cited as a major reason for adolescents having to grow up alone and neglected, which created the conditions for loneliness and depression. Some adolescents described growing up in single-parent households, as within many families, one parent is required to take on jobs in the cities or in foreign countries to meet financial needs. One adolescent shared their experience:

“I could have been an outgoing person, but both of my parents work and remain out mostly. The only time they spend at home is when waking up in the mornings and while sleeping at nights. Father has mostly been working in foreign countries. Mother’s office ends at 5 PM. Before that, I stay alone at home, and as a result got used to staying alone.” – *14-year-old depressed female (NP-036)*

Adolescents narrated that they often felt a lack of support or understanding from their parents. Living through difficult situations at home and not having the opportunity to express their feelings or feel supported exacerbated adolescents’ distress. Generational gaps between parents and adolescents were cited as causing strains in communication which created distance between parents and adolescent children as well.

“Yes, lack of love and affection from parents also can lead to depression. I felt helpless and I used to think that no one loves me.” *– 20-year-old depressed female (NP-006)*

A non-depressed adolescent also shared this view of generational communication gaps:

“The major causes for depression among adolescents are relationship issues and the other one is family. Parents don’t understand their children. It is said that parents have to behave like they are friends with children while they are in adolescence, but there is no such environment here in our society.” *– 22-year-old female with no depression (NP-007)*

Adolescents described how they witnessed fights and domestic violence among parents, or experienced emotional, verbal and physical abuse at home. These experiences were often kept to themselves and created the conditions for depression. One adolescent described the violence they witnessed at home, and how that had led them to seek out psychological counseling:

“I had spoken with my teacher recently. There was a fight between my mother and father at home. I was crying when I told her, and my teacher suggested that I take counseling. That night it was around 10 PM and we were eating. My mother was very angry that day. She confronted my father for being late. They started arguing and father started beating mother. My brother and I were there, and we started crying. My father went to the kitchen to bring a knife. He then took mother to the other room… I think of dying sometimes. Father and mother usually fight. I used to cry for entire nights and my eyes would get swollen. Father did not understand this. He was worse in the past, into drugs as well. … Father and mother talked about separation too. I didn’t want to show these things at school, always tried to smile. As I was always crying at home, I want to keep smiling at school. I do not share things with others. I only shared with my teacher recently. I wish things were a bit better at home.” - *13-year-old female recently referred to care (NP-034)*

Another adolescent who had initiated counseling on their own without their family’s knowledge shared that she did not really have a relationship with her father. She described how it affected her, and also recounted how her parents and brother used to physically beat her:

“My father was different with me from the start. Till now, I have not experienced what a father’s love is like. He used to stay far away [due to migrant work] since childhood. I want to cry when I see other people’s fathers because I also want to be with my father. Everybody says a daughter’s favorite is her father, but I can’t relate to that. My mother has a short temper and is always fighting with everyone. I cannot share my feelings openly with her. She is very strict. So, I feel bad… My mother and father were also always fighting whenever I came back home. So, I was not happy at home and used to stay outside. My elder brother was also aggressive. He used to always beat me. My father and mother both used to beat me too, so I started to feel very frightened at home.” *– 13-year-old female recently referred to care (NP-035)*

Fractious relationship dynamics with parent were discussed as triggers for slowing recovery or act as risk factors for depression relapse. When probed on what could trigger a return back to a more severe depressed state one adolescent currently undergoing treatment and in recovery shared:

“If my mother scolds me [depression could get triggered] …My mother is also a working woman. She mostly spills out her office *tensions* at home. She does that quite frequently which has been creating a lot of problems.” – *20-year-old depressed female (NP-043)*

All adult stakeholders spoke about the important role of family for adolescent mental health. One social worker shared:

“This is a busy world now. Parents may not get to spend quality time with children because they have to earn money. Adolescence is an important period where quality time with parents is needed, but it is not happening. Accordingly, adolescents might be turning elsewhere [for support]. They do not get sufficient care. There is the generation gap. There is a lack of love, guidance, and inspiration. In my opinion, these are reasons why [depression] is happening.” *– 39-year-old male social worker (NP-033)*

### Peer problems

All adolescent respondents strongly endorsed that depression could result from experiencing problems with their friends and peers. Three main aspects of peer relationships were discussed. First, adolescents shared that having unstable or unfulfilling friendships, fights amongst friends, or having difficulty establishing meaningful friendships could be major contributors to depression. One adolescent described her experience transferring to a new school and being unable to connect with her fellow students, experiencing ostracization, and how that led to depression over time:

“I was in 6th grade when I went to a boarding school. Students there were a bit older than me and as a result, I could not bond or mix with them. They never loved me and used to ‘mentally torture’ me all the time. I used to cry a lot, but I never told anyone about it…. That year was very difficult for me as I did not share things with anyone and used to keep it all inside me. Instead of defending myself from others, I have a habit of keeping things to myself and feeling sad when people say bad things to me. Also, when I was in my second year there, I had a ‘love tragedy’ – All these led me to depression.” – *20-year-old depressed female (NP-006)*

Secondly, many adolescents reported first experiencing romantic feelings during adolescence. Adolescents shared how not having a romantic partner or experiencing problems in romantic relationships could contribute to depression. One respondent shared:

“Break-ups of romantic relationships take most people into depression. The most common reason I have heard is love stories and relationships [causing depression] … Some might say, ‘don’t do this to me, don’t break up.’ Then the *tension* rises gradually. They get angry and slowly go into depression… I think the ones who fall in love deeply are the ones who go into depression.” – *13-year-old female recently referred to care (NP-034)*

Finally, adolescents strongly endorsed bullying, teasing, social exclusion, humiliation, and other negative peer interactions as major causes of depression. When asked about their depression, one adolescent recollected their experience of bullying in school:

“Since childhood, I don’t really mix well with others and prefer to stay alone. I used to speak very little. I was also bullied when I was a child because I am small (physical stature) compared to other students in the class. I was naïve and used to get scared. Everybody used to dominate over me and say: ‘You are not my friend, go away.’ So, since childhood I had no faith in friendship, and used to feel bad.” *– 13-year-old female recently referred to care (NP-035)*

Bullying was said to contribute significantly to instigating feelings of exclusion, which was connected to the overarching theme of loneliness, causing further withdrawal from friends. One respondent shared their experience:

“[Depression can happen] when bullying happens. It feels like one does not have that many friends. It feels lonely and when one feels lonely, they don’t want to interact with anyone, they want to stay introverted, they do not want to make friends. If they are like me, then they would opt to stay in a dark room, listen to music and just remain silent.” – *14-year-old depressed female (NP-036)*

Non-adolescent stakeholders also endorsed the important role of friendships and romantic relationships during adolescence and how difficulties in these relationships can contribute to depression. One social worker shared:

“Other causes [of depression] are family disputes, conflicts with their friends or family, and also because of love and ‘tragedy’ (romantic breakups). At this stage, adolescents are attracted to their opposite sex, but they do not understand [what’s happening] sometimes, and there is the perception of their family [on romantic partners] as well. Some experience ‘love tragedy’ and some get married at this stage. It is a phase where lots of things can change; some adolescents can manage, but others go through different problems that they can’t handle.” *– 42-year-old social worker (NP-001)*

### Social media

Social media as a contributor to depression was endorsed by almost all adolescent respondents. Social media was described as a space where adolescents interacted with their social circles, which then created conditions for negative aspects of peer relationships to continue beyond in-person interactions. Social media and online gaming were described to be addictive habits, often contributing to receding away from family, general social withdrawal, and reinforcing feelings of loneliness:

“It is definitely connected (social media use and depression). It forms the habit of staying alone even when you have your whole family with you and not being able to talk to them even if you want because you are now addicted to social media and mobile technology.” *– 18-year-old depressed male adolescent (NP-011)*

Other negative aspects adolescents discussed included online bullying, online relationships troubles, and the leaking of personal information or explicit photographs. These were said to be major risk factors for depression:

“I think, most of the people go into depression due to Facebook…The nude photos are uploaded after they have been Photoshopped (digitally modified). Some are being blackmailed. Some fall in love over Facebook, have break-ups and go into depression.” – *13-year-old female recently referred to care (NP-034)*

One of the major issues raised by adolescents was how social comparison with others via social media could exacerbate anxiety and depression symptoms. One respondent, who recently decided to delete their social media, shared:

“Actually, it has been 2-3 months that I stopped using Instagram. I have made a lot of changes. It is nearly 4 years that I stopped using Facebook. I used to go on Instagram before and now … I have felt a big difference. When not using Instagram, there is no comparison with others. I do not know what is happening in other people’s lives and they also have no concern about my life. When I use Instagram, I feel like that person is doing all these things, while I am staying idle. That used to trigger [feelings of sadness]. Since leaving social media, I have found a lot of changes. Social media is a triggering factor [for depression] because, in our generation, it is like an addiction.” – *20-year-old depressed female (NP-043)*

Another adolescent described how viewing social media could act as a trigger for their depression:

“I think when I see people travelling to many places in my social media newsfeed I feel a bit lonely and sometimes that triggers depression.” – *20-year-old depressed female (NP-021)*

Adolescents described how people may display an exaggerated image of happiness and success in social media which triggers stress and *tension* due to social comparison. Popularity metrics such as “likes” were also discussed as affecting confidence and decreasing self-esteem:

“The *tension* is there. Most of the time, there is a jealousy that someone got more ‘likes’ than me. About [social media] comments: it feels good if somebody has commented well but if somebody has commented negatively, it seems like it decreases self- confidence. People are now taking social media as a competition. Someone may not be happy in real life but tries to show they are happy and rich in social media. These increase *tension*.” *– 13-year-old female recently referred to care (NP-035)*

Adult stakeholders discussed a range of negative effects social media has on adolescent mental health. Phone addiction, addiction to sites like Facebook or Instagram, and compulsive online gaming were said to contribute to social withdrawal in adolescents. Other issues raised by stakeholders as being related to depression included social media misinformation, exposure to harmful people via social media groups, cyber bullying, social comparison leading to low self-esteem, blackmail, and leaking of private photographs. One psychologist shared how social media interactions can harm depressed adolescents:

“In social media, if a person posts something saying that they are having problems because of depression, some people who have positive perceptions about depression could help them. But if people start to criticize the person, it could make the situation worse… So, in my opinion, I don’t think using social media to discuss depression is a good idea. There is one case which I have seen personally. These adolescents use Instagram too much. In such social media sites, people form self-help groups, and they can really manipulate an adolescent. It can get to a state where the individual is not able to control their own emotions. Such things worsen their [depression]. I have found many such cases and had to ask them to stop using social media.” *– Female Psychologist (NP-005)*

## Discussion

In this qualitative study with Nepali youth and other stakeholders, loneliness emerged as the most salient experience of adolescent depression. Loneliness has been studied across disciplines and a recent examination [40] of its definitions suggests that it can be distilled to indicate an experience that is ultimately a function of social relationships. It has been referenced as the actual unmet need or deficit in social relations [41]. Alternately, loneliness has been described as the negative affective state attributable to a cognitive discrepancy between a person’s desired social relationships compared to what they perceive the status of those relationship to be [42]. Others have conceptualized the construct as a more fundamental human experience in response to diminishing social ties [43]. Loneliness is also related closely to the construct of social isolation but is distinct in important ways. While social isolation is an objective measure of the absence or near-absence of social relationships or frequency of contacts with others, among other objective measures, loneliness is best operationalized as a subjective emotional experience of unpleasantness due to perceived unmet needs in social relationships, as discussed above [44, 45]. Due to the inherently subjective nature of qualitative research, we determined loneliness to be the better fitting construct to label the experiences related by respondents in this study.

Five other clusters of experience were important for respondents as well: (1) sadness, low mood, and anhedonia; (2) irritability and anger; (3) somatic problems; (4) negative thoughts, rumination, and worries; and (5) suicidality. In Table 2, we present a comparison of our findings with DSM criteria and globally salient symptoms of depression from a review of qualitative studies [14]. Loneliness was universally discussed by Nepali adolescents in the current study, which was also endorsed in 60 percent of studies in Haroz and colleagues’ systematic review. Other areas of alignment between Nepali adolescents and global populations were anger and irritability, fatigue and loss of energy, appetite issues, negative thoughts, crying, and depressed mood. Nepali perspectives did not include psychomotor retardation/agitation or concentration problems, as listed in DSM criteria.

**Table 2:** Frequently mentioned aspects of depression in Nepali youth and global populations compared to the Diagnostic and Statistical Manual of Mental Disorders, Fifth Edition (DSM-5), criteria

*Current study results*	*DSM-5 Diagnostic Criteria *[11]	*Haroz *et al*. *[14]*: ‘Non-western populations’*
*Loneliness: not meeting with friends, staying silent, wanting to be alone (9/9; 100%)*	– not included in DSM-5 –	*Social isolation/ loneliness (52%)*
*Low mood: sad all the time (7/9; 78%)*	*Depressed mood*	*Depressed mood/ sadness (68%)*
*Negative self-appraisals: hopelessness, self-doubt, not able to meet other’s expectations (6/9; 67%)*	*Worthlessness or excessive or inappropriate guilt*	*Worthlessness / guilt (32%)*
*Suicidality (5/9; 56%)*	*Recurrent thoughts of death; suicidal ideation or attempt*	*Suicidal thoughts (44%)*
*Anhedonia: ‘can’t laugh like others’; not wanting to do things that one previously liked (5/9; 56%)*	*Diminished interest or pleasure in activities*	*Loss of interest (38%)*
*Irritability and anger: irritated by small things, aggression towards others; angry outbursts (5/9; 56%)*	*Irritable mood (included as an alternative to depressed mood for children and adolescents in DSM-5)*	*Anger (36%)*
*Crying a lot (4/9, 44%)*	– not included in DSM-5 –	*Crying a lot (46%)*
*Sleep disturbance: sleepiness throughout the day; (4/9; 44%)*	*Insomnia or hypersomnia*	*Problems with sleep (63%)*
*Stress or Tension (3/9, 33%)*	– not included in DSM-5 –	– not reported –
*Thinking too much (3/9, 33%)*	– not included in DSM-5 –	*Thinking too much (39%)*
*Appetite: loss of appetite (2/9; 22%)*	*Weight loss or weight gain; decrease or increase in appetite*	*Appetite/ weight problems (50%)*
*Chronic Fatigue: low energy to do things (2/9; 22%)*	*Fatigue or loss of energy*	*Fatigue/ loss of energy (63%)*
*Headaches (1/9; 11%)*	– not included in DSM-5 –	*Headaches (45%)*
– not reported –	*Diminished ability to think or concentrate; indecisiveness*	– not reported –
– not reported –	*Psychomotor agitation or retardation*	– not reported –

In Nepal, loneliness was described to be interwoven through all other aspects of depression by respondents. For many adolescents, loneliness did not necessarily mean the absence of meaningful relationships; some adolescents described having meaning friendships, but an inability to engage in those relationships because of the depression. Overall, feelings of loneliness were reported despite having good friends and supportive family members. Some adolescents commented that withdrawing into oneself due to depression slowly led to abandonment by friends. The strongest reactions to depression were a desire to be left alone and not wanting to open up to others and share their feelings. Adolescents described sadness, negative self-appraisals, self-doubt, and anger all combining together to contribute to this powerful desire to be left alone. Loneliness was described to work in a mutually reinforcing vicious cycle with mood and cognitive aspects of depression, amplifying the experiences of sadness, negative thoughts, and anger.

Nepal can putatively be considered a collectivist society [46], where the cultural system values a socio-centric interdependent view of the self in relation to others, and where the group is elevated over the individual. Accordingly, experiences which result in diminishing actual or perceived group membership may be more salient for depression in this cultural context. This could potentially be a macro-level explanatory factor of why loneliness emerged as such an important experience for depressed adolescents in Nepal. This is supported by another recent qualitative study of adolescent depression in rural Nepal, which applied an interpersonal therapy model of depression to interpret causation [22]. That study found that most adolescent experiences of depression could be attributable to interpersonal disputes. Supporting our findings, the isolation was also commonly described despite the availability of physical proximity to others and extensive family and community social networks. Taken together, these findings suggest that loneliness and isolation are tied to inability to engage with social networks rather than a lack of available networks.

Furthermore, it may also be plausible that loneliness during adolescence, a period characterized by the crucial drive to form bonds with peers [2], may transcend cultural concepts of identity and represent a more universal aspect of adolescent depression related to disconnection from social groups. A recent qualitative study of adolescent depression in Brazil found that depression was characterized by “a feeling of detachment from others resulting from the sensation that usual interactions did not have the same meaning as before.” [20] It is also interesting that in a recent meta-synthesis of qualitative studies on adolescent depression from high-income countries (HIC), loneliness was found to be the most salient aspect of experience as well [47]. The findings from that review align with results of this current study in some key areas. Adolescents from HIC shared similar strong feelings of wanting to be alone, and the inability or unwillingness to share their problems, which mirror the experiences of Nepali and Brazilian youth. Achterbergh and colleagues [47] also find that adolescents from HIC retain a desire to connect to others, especially friends, despite their feelings of loneliness. Although some Nepali adolescents spoke of desiring better relationships with their parents, most Nepali and Brazilian youth described avoidance of peer engagements. Finally, HIC adolescents also described feelings of loneliness which led to depression, which in turn increased loneliness, in a vicious cycle—which reflect the experience of Nepali adolescents in the current study as well.

Additionally, loneliness may also be connected to the concept of ‘thwarted belonginess’—a central construct of the interpersonal theory of suicidal behavior, which has been validated amongst adolescents [48]. Thwarted belonginess is characterized as the distressful mental state that emerges when the fundamental need for connectedness is not fulfilled [49]. In fact, loneliness has indeed been found to be connected with suicidality in some LMIC settings. In a study of adolescents from Thailand, Philippines, and Taiwan, loneliness was significantly associated with a suicide attempt [50]. Additionally, controlling for loneliness substantially weakened the association between hopelessness and suicide attempts. In the current study, loneliness was invoked by Nepali adolescents as leading to suicidal thoughts or attempts, and not exclusively due to related hopelessness.

Descriptions of symptom clusters by Nepali adolescents were also noteworthy because these were rarely discussed as separate components, but as interdependent and presenting in cascading causal chains. For example, sleep disturbances and appetite loss were said to result in low energy, and over time, low energy and fatigue were described to lead to tendencies of losing interest in normal activities and social withdrawal (similar to anhedonia). Flashes of extreme anger were described to precede and trigger thoughts and acts of self-harm and suicide. Suicidality was also described to be rooted in negative beliefs about oneself and other cognitive distortions, and feelings of sadness and loneliness. These qualitative perspectives may be better explained by the network theory of depression where symptoms of depression are conceptualized as having associative relationships (e.g., symptoms such as sleep disruptions and energy are not independent of each other; rather, these are connected where disturbed sleep causes loss of energy) and are constitutive of the condition (i.e., the symptoms as a collective are the condition), rather than proxy indicators of an unobserved latent condition [51]. Indeed, in a network analysis of depression among Brazilian youth (a different component of the IDEA research study), the feeling of loneliness was found to be one of the most central and salient aspects in a network comprising both DSM and non-DSM symptoms [52].

The use of idioms of distress such as *tension* or the English word ‘depression’ to communicate both depressive symptoms and normal negative affect reflects an evolving linguistic landscape in how young people understand and communicate internal thoughts and feelings. These findings are important for stakeholders such as parents, counselors, primary healthcare providers and teachers, who are well positioned to detect changes in adolescent behaviors early. Such narratives could be indicative of worsening mental health and necessitate referral to specialist providers for assessment. For mental healthcare providers, such cases would warrant deeper investigation to probe for underlying disorder and should not be dismissed as trivial accounts of non-pathological distress. On the other hand, it is vital that complaints of *tension* are not assumed acritically or reflexively as a clinical condition, i.e., *tension* should not be taken to be as synonymous with depressive disorder. While some forms of *tension* may be indicating mild distress, other forms of *tension* may be more comparable to depression and clinical psychopathology, and therefore, careful assessment is necessary. Interestingly, recently conducted research in Bangladesh with young male slum residents (ages 18–29; mean age of 19 years) found *tension* to be used by respondents to communicate a similar range of distress, from nonpathological psychological complaints to severe symptoms comparable to depressive disorder [53, 54].

Adolescents and other stakeholders raised the important role of parental and family issues, peer relationships, and social media in causing depression. While family issues and peer relationships are established risk factors for depression, social media presents a developing space with both risks and opportunities [55]. A recent systematic review of studies from both HIC and LMIC found correlations between social media use and depression, anxiety, and general psychological morbidity in adolescents [56].

The findings of this study indicate that interventions addressing loneliness and quality of social support could be developed and tested in Nepal to address adolescent depression. As loneliness is reflective of dysfunctional interpersonal relationships, one potential candidate intervention for depressed adolescents or youth afflicted with loneliness could be Interpersonal Therapy (IPT). A recently conducted cultural adaptation of IPT in rural Nepal found that including activities that focused on strengthening relationships between group members were a key component of IPT delivered in schools [57]. This likely indicates that addressing loneliness may be an integral component of depression interventions for young people in Nepal. Other salient factors of IPT adaptation included the incorporation of locally used terms for depression. As respondents frequently referred to *tension* in the current study, a similar approach can be explored for interventions developed for this population. Indeed, “*heart-mind tension*” was the central cultural construct of a recently conducted adaptation of Problem Management Plus intervention in Nepal as well [58]. Additionally, examining loneliness as a risk factor for developing depression using prospective study designs can be a valuable area for future research. Finally, early interventions addressing loneliness in youth at-risk for depression could be tested for effectiveness in preventing the future onset of depression as well.

## Limitations

There are several limitations of this study. First, the study participants were recruited from the greater Kathmandu area, which comprised a distinctly urban sample. This limits the study’s ability to accurately reflect the contextual heterogeneity between rural and urban Nepal. This difference is important considering marked differences in context and experiences induced by rapid urbanization in Kathmandu city compared to the relatively slower agrarian lifestyle in rural areas. Secondly, the adolescent sample was comprised of more female participants due to recruitment dynamics, and therefore the perspectives are more heavily informed by female views and experiences. Accordingly, caution needs to be exercised in transferability of the results.

## Conclusion

In this qualitative study of adolescent depression, a range of distressful emotions and experiences were described by adolescents and other stakeholders in Nepal. Of these, subjective feelings of loneliness were considered to be the most important and emerged as connected to all other aspects of the lived experience of depression. These narratives were synthesized and presented in a framework of adolescent depression in Nepal, exhibiting the interconnections of symptoms that comprise the experience of depression, and the key causal clusters behind the condition. Further research utilizing statistical methods and longitudinal designs are necessary to complement the qualitative perspectives identified in this study. Interventions addressing loneliness can be of value to ease suffering and lessen the impact of depression for millions of adolescents living in Nepal.

## Supplementary Information

**Additional file 1:** Qualitative Guidelines.

**Additional file 2:** Authors’ background and training.

**Additional file 3:** COREQ (COnsolidated criteria for REporting Qualitative research) Checklist.

## Data Availability

The datasets used and/or analyzed during the current study are available from the corresponding author on reasonable request.
