# Supplementary material for: Adolescent perspectives on depression as a disease of loneliness: a qualitative study with youth and other stakeholders in urban Nepal
Source: Child Adolesc Psychiatry Ment Health. 2022 Jun 23;16:51. doi: 10.1186/s13034-022-00481-y (PMC9229752; doi:10.1186/s13034-022-00481-y)
Supplement: Supplementary file 1 — Additional file 1: Qualitative Guidelines. [file 13034_2022_481_MOESM1_ESM.docx]

| **KII Guidelines** | | | **Respondents** |
| --- | --- | --- | --- |
| **Q. No.** | **Main Questions** | **Probe questions** |  |
| *NOTE: Remind respondent that this study is happening in 4 countries, and local concepts should be described in detail. These could include any local metaphors, cultural references (movies, characters, politicians, stories, etc.), abbreviations (CPS/NHS), ways of speaking (“love tragedy”), etc.* *Interviewer should be attentive about this during the interview and probe and ask for detail if respondent uses local terms. You can provide an example from your country’s context and tell participants that it is important that such local terms or concepts need to be explained in detail so the international researchers working on this study can understand what they are trying to communicate.*  Introduction: I would first like to know a little about your personal background and what aspects of your work or training involve adolescents and mental health issues. | | | |
| **1.** | **Introduction (Grand Tour) Questions** |  |  |
| **1a** | Please, tell me about your professional background and what aspects involve adolescents? | *Probe: If the participant does not mention issues related to mental health, ask a probe question about “and what aspects of your professional training and current work involve mental health-related topics?”* | Teachers, Social workers, policy makers, care providers; |
| **1b** | I would like to learn a little about your life, could you tell me what an average day is like for you? (What do you do in the mornings, during the day, and in the evening?) | *If needed, Probe: About school, about family, about friends (how many; best friend; what you do together? Etc.)*  *[Note – these are only to be asked if the adolescent is hesitant about opening up initially]* | Adolescent |
| **1c** | I would like to start by learning a little about your family. Would you mind telling me a little about your household (how many members in the household, who lives there, how many children, their ages)? |  | Parent |
| **2.** | **Healthy Adolescent Development** |  |  |
| **2.1** | In your opinion, which age group is defined as adolescence? |  | All |
| **2.2** | For you, what are the unique aspects of this stage of life? | *Probe: How would you describe adolescence?*  *Probe: What have been the changes in relationships, activities, societal expectations, etc. in your life (in your child’s life), (in the lives of adolescents you work with)?* | All |
| **3.** | **Depression in adolescence** |  |  |
| **3.1** | Could you give me an example of an adolescent in your life (friend, relative, client) whom you would consider to have good mental health and wellbeing? | *What are their characteristics that demonstrate good mental health and wellbeing?* | All |
| **3.2a** | Now could you give me an example of an adolescent in your life (friend, relative, client) whom you would consider to have had some mental health difficulties and possibly be depressed? | *What are their characteristics that demonstrate that this person may have been depressed?* | Teachers, Social workers, policy makers, care providers; |
| **3.3a** | Thinking about adolescents that you know whom have had mental health problems and may be depressed, what do you think distinguishes that depression from normal ‘sadness’? | *Probe: Are there local terms that are used for depression?*  *Probe: For a depressed person, they may be feeling many things on the inside but showing only a few things on the outside – can you give me an example from your experience of an adolescent who was going through something like this?*  *Probe: physical sign/ symptoms; mental/ emotional symptoms* | Teachers, Social workers, policy makers, care providers; |
| **3.4a** | Thinking about the adolescents that you know whom have good mental wellbeing, can you describe some of the key factors in their lives that contribute to their wellbeing? | *Probes: What about their relationships, their schools, there families, other factors…?* | Teachers, Social workers, policy makers, care providers; |
| **3.5a** | Thinking about the adolescents in your life who have had some mental health problems and may be depressed, what do you think contributed to their depression? | *Probes (use as necessary):*  *What are some issues that are present in the home environment? How about at school? What about in the community? Any other?* | Teachers, Social workers, policy makers, care providers; |
| **3.6a** | Gender differences *[if the respondent has not mentioned gender differences, then ask this question]*.  We have just been talking about mental wellbeing and depression among adolescents. From what you know, is the outward expression of wellbeing or depression different between boys and girls? Can you give an example? | *Probe: What about the internal experience of depression? Can you give an example?*  *Probe: Do you think there are differences in risk factors between boys and girls? How so?* | Teachers, Social workers, policy makers, care providers; |
| **3.7a** | Thinking of adolescents whom you know who may have depression, what are some of the impacts of depression on their lives? Please give examples or tell me the story of how depression impacted an adolescent’s life? | *Probe (Only if necessary, and not all are needed):*  *On physical health or mental health*  *At individual or home level*  *At school level*  *At community level / social life*  *At work*  *Others* | Teachers, Social workers, policy makers, care providers; |
| **3.2b** | I would like to ask some questions now about your life and experiences, feel free to let me know if you don’t want to answer any of the questions. First, could you tell me about a time in your life when you encountered a difficulty and felt distressed? How did you deal with it? |  | Adolescent |
| **3.3b** | **For both healthy & depressed adolescents:**  Have there been times in your life when you felt depressed? How could you tell it was depression and not just normal sadness?  **For healthy adolescent if they respond that they are not depressed:**  Can you think of an adolescent friend, family member, or school mate, who may have or is going through depression? How could you tell it was depression and not normal sadness? | *IMPORTANT – Probe with all of these questions:*  *How would you describe the experience of depression to someone else who hasn’t had it before?*  *When you experienced depression, how did you describe it to your parents, teachers, or friends?*  *What exact words/phrases did you use?* | Adolescent |
| **3.4b** | **For depressed adolescent:**  In your life, what are the things that have prevented you from becoming depressed, or helped you feel better when you are depressed?  **For healthy adolescent if they responded that they are not depressed:**  Thinking of the adolescent you know who has/had depression, what are some of the things that can help them not be depressed, or help them feel better when they are depressed? |  | Adolescent |
| **3.5b** | **For depressed adolescent:**  In your life, what are the things that are most likely to trigger depression? Can you give me some examples?  **For healthy adolescent if they responded that they are not depressed:**  Thinking of the adolescent you know, what are the things that are most likely to trigger depression? Can you give me some examples? |  | Adolescent |
| **3.2c** | I would like to ask some questions now about your adolescents’ life and experiences, feel free to let me know if you don’t want to answer any of the questions. First, could you tell me about a time in her/his life when he/she encountered a difficulty and felt distressed? How did she/he deal with it? |  | Parent |
| **3.3c** | Have there been times in your adolescent’s life when she/he felt depressed? How could she/he be experiencing depression and not just normal sadness? | *How did your adolescent describe the experience of depression to you?* | Parent |
| **3.4c** | In your adolescent’s life, what are the things that help prevent depression? | *What helps her/him feel better when she/he is depressed?* | Parent |
| **3.5c** | In your adolescent’s life, what are the things that are most likely to trigger depression? Can you give me some examples? | *Are there things from early in life that put them at risk of depression?* | Parent |
| **3.6** | *Only for Nepal:*  Can you tell describe the difference between ‘*tension’* and depression? |  | All |
| **4** | **Identification of depression in adolescence** |  |  |
| **4.1a** | From your personal or professional experience, can you give an example of when depression was identified early, and the adolescent was given support or taken for care? | *How was the depression recognized early?* | Teachers, Social workers, policy makers, care providers; |
| **4.2a** | Based on you experiences, what are the major barriers for identification of depression among adolescents? Please give some examples. | *Probe about stigma*  *Note: Ask for* ***specific examples*** *of barriers.*  *At individual or home level*  *At school level*  *At community level*  *At non-mental health service provider level*  *At service provider level* | Teachers, Social workers, policy makers, care providers; |
| **4.3a** | From our discussion so far, it would seem very important to identify depression early in adolescents. In your opinion, how can we better identify depression among adolescents in <insert country>?  Do we need to provide training for identification of depression at each level? | *Probe:*  *At individual or home level*  *At school level*  *At community level*  *At non-mental health service provider level*  *At service provider level* | Teachers, Social workers, policy makers, care providers; |
| **4.1b** | **For depressed adolescent:**  When did you get treatment or professional care for your depression? What led to getting professional care?  **For healthy adolescent:**  Thinking of the adolescent you know, do you know anything about if they are getting treatment currently or received treatment in the past for their depression? When did they get care? What led to getting professional care? |  | Adolescent |
| **4.2b** | **For depressed adolescent:**  Are there things that you think could have helped others recognize the risk factors for depression or early symptoms in your life?  **For healthy adolescent:**  Thinking about the adolescent you know, are there things that you think could have helped others recognize the risk factors for depression or early symptoms in their life? |  | Adolescent |
| **4.3b** | What do you think could be done to help others recognize the risk factors for depression early and then do things to prevent it? |  | Adolescent |
| **4.1c** | When did your adolescent get treatment or professional care for depression? What led to getting professional care? | *Were there certain symptoms or problems that led to getting care?* | Parent |
| **4.2c** | Are there things that you think could have helped recognizing the risk factors for depression before it started? |  | Parent |
| **4.3c** | What do you think could be done to help others recognize the risk factors for depression early and then do things to prevent it? |  | Parent |
| **5.** | **Risk Calculator** |  |  |
|  | **Introduction**  *Consider this sample introductory language for adolescents/parents/non-service providers:*  Since we just talked about how someone could identify an adolescent with depression, we are now going to discuss how we could potentially predict if an adolescent might have a higher likelihood of developing depression in the future. Screening tools for depression can tell us if someone is currently, **at the present time**, at risk of having depression. However, a risk calculator is different in a small but important way – it can tell us if a person is at risk of developing depression, **in the future**. Early detection and prevention of depression can help adolescents and their support networks become more aware of their mental health, seek access to services sooner, and subsequently prevent depression or begin treatment in earlier stages of depression.  *Consider this sample introductory language for service providers:*  Since we just discussed how one might identify an adolescent with depression, we would now like to discuss how one might identify an adolescent who is at high risk for developing depression in the future. For example, in some countries they now have a risk calculator, or a questionnaire, that individuals can fill out to determine whether they might be at a higher risk for developing diabetes in the future. Screening tools for depression can tell us if someone is currently, **at the present time**, at risk of having depression. However, a risk calculator is different in a small but important way – it can tell us if a person is at risk of developing depression, **in the future**.  *Sample risk calculator explanation language for all participants:*  With a risk predictor for depression, we hope to be able to quickly identify adolescents who may be at higher risk and intervene to prevent them from developing depression. We have been working on a tool to help identify those adolescents who may be high risk. Screening tools for depression can tell us if someone is currently, **at the present time**, at risk of having depression. However, a risk calculator is different in a small but important way – it can be used to identify adolescents who have few or no symptoms now but may have a higher likelihood of developing depression in the future.  *Open up “Risk Calculator Prototype” with Participant and show them the first page:*  This risk calculator is formatted as a questionnaire that asks questions related to potential risk factors for depression among adolescents. The current prototype is for adolescents to answer the questions themselves; however, we will ask you after you see the tool if you think that others (e.g., parents, teachers, health workers) could also use the tool in some way. I would like to walk through it with you, and I ask that you provide feedback on the calculator when we are finished. For this exercise, you do not have to answer the question as yourself; You may think of another person or answer as an imaginary person. This is only to familiarize you with the questions so far and to help us improve the calculator with any comments or suggestions you may have. Since it is only a prototype, there will be no actual score calculated at the end. You will be able to see the results for both “high risk” and “low risk” responses at the conclusion of the questions.  *[NOTES: All questions must be answered, i.e. no “don’t know” or “prefer not to answer” responses; No actual score will be calculated at the end.]*  *After completing the risk calculator and demonstrating both the “high risk” outcome and “low risk” outcome page, ask the following questions and use Probes when needed:* | | |
| **5.1** | What is your initial response to the risk calculator? | *Probe: Length of questionnaire, acceptability/appropriateness of questions, usability* | All |
| **5.2** | Who do you see using this calculator, and where or when would they use it? | *Probe for adolescents: What are the potential benefits of using a calculator like this?*  *Probe: If the participants asks how the risk calculator is different from PHQ, CESD, MFQ, etc., then explain again that unlike screening tools which detect risk of depression at the present time, the risk calculator predicts risk of developing depression in the future, and then ask the participant if she/he sees the risk calculator as having use in addition to screening type tools.* | All |
| **5.3** | Do you foresee any challenges for using this questionnaire? If so, how might we address them? | *Probe: What are the potential benefits of using a calculator like this?*  *Probe: Are there any potential negative consequences of using something like this?*  *Probe: What solutions or changes would you recommend for these challenges?* | All |
| **5.4** | What additional resources or instructions would need to go along with such a calculator? | *Probe: What additional information would you like to know for someone who was high risk? For someone who was low risk?* | All |
| **6** | **Management of Depression** |  |  |
| **6.1** | Will you tell me the story about an adolescent you know (or ‘your story’ if interview is with adolescent), and their coping habits? It could be both positive coping and/or negative coping methods. | *NOTE: If respondent’s story is about positive coping only or negative coping only, ask about the opposite.*  *Are there any differences comparing girls vs. boys?* | All |
| **6.2a** | Let’s discuss treatment and care for adolescents with depression now. Will you share your thoughts on the current strategies used in <insert country> for the management of depression in adolescents? | *Probe: What strategies helped, did not help, or made things worse?* | Teachers, Social workers, policy makers, care providers; |
| **6.3** | Can you tell me the story of an adolescent you know (you helped; you treated) and how their depression was managed/treated? | *Probe: What could be done to help manage/treat depression better for adolescents?*  *Probe: Give me an example from (choose most appropriate level):*  *At individual or home level, at school level, at community level, at service-provider level*  Do we need to provide training for management of depression? What type of training and for whom?  *Note: If they previously mentioned substance abuse, probe on how we can better manage depression and coping with substance use* | Teachers, Social workers, policy makers, care providers; |
| **6.4a** | Thinking back to adolescents you know who currently have depression or had it in the past, can you tell me some examples of different difficulties they faced accessing treatment or during their treatment? | *Probe (only if appropriate):*  *At individual or home level; At school level; At community level; At non-mental health service provider level; At service provider level*  ***Probe:*** What solutions would you propose to overcome these barriers? | Teachers, Social workers, policy makers, care providers; |
| **6.5** | As a provider, are there specific guidelines available for managing adolescent depression at your institution? | *For example, are you using the same format for adolescents that you would use for adults?*  *Do you think it’s important to have different guidelines for adolescents vs. adult care for depression or general mental health problems?* | **Service Providers** **ONLY** |
| **6.2b** | **For depressed adolescents only:**  Can you tell me the story of how your treatment has been going so far? | ***Note****: This question is for adolescents to speak of their professional care.* | Adolescent |
| **6.3b** | **For depressed adolescents:**  What are some ways that your friends, family, teachers, etc. have helped you (or an adolescent you know) to manage depression? Give me an example.  **For healthy adolescents:**  Thinking of the adolescent we have been talking about, what are some of the ways that you, or their friends, family, teachers, etc. have helped them to manage depression? Give me an example. | *Probe: What strategies helped, did not help, or made things worse?*  *Probe: What do you wish they had done/could have done better?*  *Give me an example from (choose most appropriate level):*  *At individual or home level, at school level, at community level, at non-mental health service provider level, at service-provider level* | Adolescent |
| **6.4b** | **For depressed adolescents:**  What are some of the biggest challenges you faced in managing your depression? Give me an example.  **For healthy adolescents:**  For the adolescent you know, what do you think are some of the biggest challenges they have faced or may face in managing their depression? Can you give an example if you have one? | *Note: Ask for* ***specific examples or stories***  *Probe (only if appropriate): At individual or home level; At school level; At community level; At non-mental health service provider level; At service provider level*  *Probe: What solutions would you propose to overcome these barriers?* | Adolescent |
| **6.2c** | What are some of the ways that you have helped your adolescent to manage their depression? | *Probe: What strategies helped, did not help, or made things worse?*  *Probe: What additional resources do you think could help better manage your child’s depression?*  *Give me an example from (choose most appropriate level):*  *At individual or home level, at school level, at community level, at non-mental health service provider level, at service-provider level*  *Note: If they previously mentioned substance abuse, probe on how we can better manage depression and coping with substance use* | Parent |
| **6.4c** | What are some challenges, obstacles, or barriers that have hindered your ability to help your adolescent manage their depression? | *Note: Ask for* ***specific examples or stories***  *Probe (only if appropriate): At individual or home level; At school level; At community level; At non-mental health service provider level; At service provider level*  *Probe: What solutions would you propose to overcome these barriers?* | Parent |
| **7.** | **Behavior towards depressed adolescent (Stigma)** |  |  |
| **7.1a** | Can you give me an example of an adolescent or a teenager whom you thought was treated differently by their peers, family, or members of the society because of their depression? | *Probe: How do people in general society behave towards adolescents with depression?*  *Probe for specific details: Positive attitude/ behavior; negative attitude/ behavior (e.g. stigmatizing; ask to share case study, if any), potential causes* | Teachers, Social workers, policy makers, care providers; |
| **7.2a** | How did you or others try to address those attitudes/behaviors, or what do you wish could have been done to address that stigma? | *Probe for: anti-stigma reduction program; who needs to be prioritized for the program; how can we measure effectiveness of anti-stigma reduction program* | Teachers, Social workers, policy makers, care providers; |
| **7.3** | Have you had any specific training or coursework on the concept of stigma towards depression? Any training or coursework decreasing stigma? |  | **Providers, teachers ONLY** |
| **7.1b** | Can you think of a time when either you or an adolescent you know were/was treated differently by other people because of your/their depression? | *Probe for specific details: Positive attitude/ behavior; negative attitude/ behavior (e.g. stigmatizing; ask to share case study, if any), potential causes* | Adolescent |
| **7.2b** | What do you wish other people knew or understood about depression? | *How could we help others understand/change their behaviors?*  *If respondent says that we can “raise awareness” – ask for specific example of how it should be done, or if they have seen an example* | Adolescent |
| **7.1c** | Can you think of a time when you, your family, or your adolescent was treated differently by other people because of their depression? | *Probe for specific details: Positive attitude/ behavior; negative attitude/ behavior (e.g. stigmatizing; ask to share case study, if any), potential causes* | Parent |
| **7.2c** | What do you wish other people knew or understood about your adolescent’s depression? | *How could we help others understand/change their behaviors?*  *If respondent says that we can “raise awareness” – ask for specific example of how it should be done; or if they have seen an example* | Parent |
| **8.** | **Mobile and Information Technology** |  |  |
| **8.1** | Many adolescents these days spend a lot of time on the internet and mobile phones. They may spend a lot of time on social media like Facebook, Instagram, twitter, etc.  What has been your experience with social media in terms of adolescent mental health? | *Get specific stories or examples!*  *Probe (definitely use this probe): Let’s talk specifically about social media/cellphones and* ***bullying*** *– What are your thoughts on that? [Be sure to probe about Bullying]* | All |
| **8.2** | Can you tell me your thoughts on social media use and mobile phones, etc. and adolescent depression? | *How is it helpful? How is it potentially hurtful?*  *If respondent says that we can “raise awareness” – ask for specific example of how it should be done; or if they have seen an example* | All |
| **8.3** | Is it possible to use these social media and mobile phone technology to identify adolescents with depression? | *Probe for specific example or story* | All |
| **8.4** | How can social media and mobile phone technology be used to develop solutions for managing depression for adolescents? | *Probe for specific example or story* | All |
| **9.** | **Policy and Health System Strengthening** |  |  |
| **9.1** | How can the health system in your country be strengthened for comprehensive management of depression among adolescents? | *Probe: Resources, coverage, task shifting, training* | Teachers, Social workers, policy makers, care providers; |
| **9.2** | If a mental health policy was created for a school, what components should it contain? Will it be acceptable to implement such policies in schools? |  | All |
