# Supplementary material for: Adolescent perspectives on depression as a disease of loneliness: a qualitative study with youth and other stakeholders in urban Nepal
Source: Child Adolesc Psychiatry Ment Health. 2022 Jun 23;16:51. doi: 10.1186/s13034-022-00481-y (PMC9229752; doi:10.1186/s13034-022-00481-y)
Supplement: Supplementary file 2 — Additional file 2: Authors’ background and training. [file 13034_2022_481_MOESM2_ESM.docx]

**Additional file 2: Authors’ background and training**

1. **Syed Shabab Wahid, DrPH**: Dr. Wahid is Assistant Professor at the Department of International Health, Georgetown University. He is trained in global health with expertise in mixed-methods research. Dr. Wahid is an experienced global mental health researcher, with specialization in multi-site qualitative studies in transcultural psychiatry and global mental health.
2. **Katherine Ottman, MSc.**: Ms. Ottman has a background in anthropology and global health, and is a trained global mental health researcher with expertise in transcultural research methods.
3. **Jyoti Bohara**: Ms. Bohara is an experienced Nepali researcher with experience in conducting transcultural research in the Nepali context, and with adolescent populations particularly.
4. **Vibha Neupane**: Ms. Neupane is an experienced Nepali researcher with expertise in conducting transcultural research in the Nepali context, and with adolescent populations particularly.
5. **Helen L. Fisher, PhD**: Dr. Fisher is Professor of Developmental Psychopathology at the Institute of Psychiatry, Psychology and Neuroscience (IoPPN), King’s College London, UK. She has a strong interdisciplinary background in psychology, social psychiatry, epidemiology, genetics, and epigenetics. Her multidisciplinary programme of research focuses on the role of social, psychological, biological, and wider environmental factors in the development, course, and prevention of mental health problems in children, adolescents, and young adults.
6. **Christian Kieling, MD, PhD**: Dr. Kieling is Associate Professor of Child & Adolescent Psychiatry at the School of Medicine, Universidade Federal do Rio Grande do Sul (UFRGS). He directs the Child & Adolescent Depression Program (ProDIA) at the Hospital de Clinicas de Porto Alegre (HCPA), Brazil. Dr Kieling is a UK Academy of Medical Sciences Newton Advanced Fellow, and a Conselho Nacional de Desenvolvimento Científico e Tecnológico (CNPq, Brazil) researcher.
7. **Valeria Mondelli, MD, PhD**: Dr. Mondelli is a Clinical Reader in Psychoneuroimmunology at the IoPPN, King’s College London, and a Liaison Consultant Psychiatrist at King’s College Hospital. Her research interests focus on the interplay between physical and mental health and on the role of biological systems involved in the stress response in the pathogenesis of psychiatric disorders. With Dr Kieling, she leads a global mental health consortium aiming to identify predictors of depression early in adolescence (the IDEA project).
8. **Brandon A. Kohrt, MD, PhD**: Dr. Kohrt is an anthropologist and psychiatrist, and Professor of Psychiatry and Global Health and Director of the Division of Global Mental Health. Dr. Kohrt is a global expert in transcultural psychiatry and global mental health.
9. **Kamal Gautam, MD**: Dr. Gautam is a trained psychiatrist and senior Nepali researcher specializing in psychiatric research and transcultural methods with decades of mental health research experience in Nepal.
